# 3D anatomical model for teaching canine lumbosacral epidural anesthesia^1^

**DOI:** 10.1590/s0102-865020200060000008

**Published:** 2020-07-13

**Authors:** Eduardo Cavalcante das Neves, Charles Pelizzari, Romulo Silva de Oliveira, Siham Kassab, Kleber dos Anjos Lucas, Yuri Karaccas de Carvalho

**Affiliations:** I MSc, Postgraduate Program in Health and Animal Production, Universidade Federal do Acre (UFAC), Rio Branco-AC, Brazil. Acquisition, analysis and interpretation of data; manuscript preparation and writing.; II DSc, Veterinary Medicine Teaching and Research Unit, UFAC, Rio Branco-AC, Brazil. Technical procedures.; III MSc, Veterinary Medicine Teaching and Research Unit, UFAC, Rio Branco-AC, Brazil. Technical procedures.; IV MD, UFAC, Rio Branco-AC, Brazil. Scientific and intellectual content of the study.; V DSc, Center for Biological and Natural Sciences, UFAC, Rio Branco-AC, Brazil. Manuscript writing, critical revision, final approval.

**Keywords:** Anesthesia, Epidural, Lumbosacral Region, Learning, Printing, Three-Dimensional

## Abstract

**Purpose:**

To develop a 3D anatomical model for teaching canine epidural anesthesia (3DMEA) and to assess its efficacy for teaching and learning prior to the use of live animals.

**Methods:**

The creation of 3DMEA was based on 3D optical scanning and 3D printing of canine bone pieces of the fifth to the seventh lumbar vertebrae, sacrum and pelvis. A total of 20 male dogs were scheduled for castration. 20 veterinary students watched a video showing epidural anesthesia in dogs before the clinical attempt and were assigned to control or 3DMEA groups. Students in the 3DMEA group trained in the model after the video. For the clinical trial, the epidural procedure was performed by students under the veterinary supervision. When observed the absence of response to nociceptive stimuli, the epidural was considered successful. Then, all students answered a questionnaire evaluating the main difficulty founded in the technique and its degree of difficulty.

**Results:**

The 3DMEA group reported a lower degree of difficulty to perform the epidural anesthesia technique when compared with the control group (p=0.0037). The 3DMEA reproduced the anatomical structures, allowing the perception of the distance of needle in relation to the iliac prominences during epidural anesthesia. Its mobility allowed simulation of the animal in standing position and sternal recumbency.

**Conclusion:**

The use of 3DMEA demonstrated greater efficacy in the execution of the technique, being effective in the teaching and learning process before the epidural anesthesia in live animals.

## Introduction

The epidural lumbosacral anesthesia is a practical technique that, if performed correctly, can be a very useful complementary method to general anesthesia that promotes pre- and post-operative analgesia. The technique is considered safe and induces minimal cardiovascular and pulmonary changes^1,2^. The palpation of anatomical references such as the iliac prominences and the spinal process of the last lumbar vertebra (L7) in the dog are indispensable for performing the procedure^3^. Factors such as obesity and incorrect positioning during the technique make it difficult or impossible to perform it^4^.

This technique is currently taught through lectures and demonstrations in veterinary medicine colleges; in some cases, students practice it in live animals under supervision^5^. Despite the technique’s importance and effectiveness, it is known that the conduction of practice classes with live animals is limited by the reduced number of animals in which it can be practiced and student insecurity and discomfort performing it in this animals^6^.

In human medicine, simulators are used to improve training in anesthesia^7^. Most try to simulate the sensation of penetration of ligamentum flavum and loss of resistance of the syringe plunger during injection of the anesthetic. These commercial models are usually expensive, and the resistance sensation of ligamentum flavum is rapidly lost, making the search of homemade models an accessible alternative^10,11^.

The use of simple and even handcrafted simulators for training students to perform the supervised technique, allows them a more realistic experience in a safe and controlled environment and reduces the risks of complications during the supervised technique^5^.

Hence, 3D printing has been presented as an innovative technology capable of favoring the direct correlation with the real anatomy in a sufficiently detailed way to provide an alternative to the use of animals and with great potential to provide a source of high-quality teaching materials^12^.

Three-dimensional technology has been used in veterinary medicine in the manufacture of implants and prostheses^13^, in medical clinics^14^, for diagnosis^15^, for surgical planning^16^, for reproduction and for teaching. The latter includes studies of anatomy^17^, clinical^18^, diagnostic imaging^19^, and surgery^20^. Despite its use in several educational areas, studies in the area of veterinary anesthesiology have not yet been reported.

Would an epidural anesthesia 3D model help the students’ training process in performing epidural anesthesia in dogs? This study aimed to develop a 3D anatomical model for the teaching of canine lumbosacral epidural anesthesia (3DMEA) and to evaluate its effectiveness in the teaching and learning process prior to its use in live animals.

## Methods

Experimental protocol was approved by the Animal Research Ethics Committee of Universidade Federal do Acre (CEUA-UFAC), protocol number 27/2018.

### *Preparation of the 3DMEA*

The 3DMEA was based on the scanning of only selected bone parts of the fifth to the seventh lumbar vertebrae (L5–L7), sacrum, and pelvis from the cadaver of a medium-sized healthy dog. The bones were digitized by a 3D optical light scanner that did the capture of images in 360 degrees for about one hour, in which its result served as the basis for modeling the 3D prototype to be printed. The generated images were saved in an .STL file format and later edited through the 3D Autodesk Meshmixer creation and editing program (Autodesk Inc., CA, USA) to perform modeling and fix any scanning flaws.

The prototype was built in the 3D printer (UP 3D Mini® - Beijing Tiertime Technology Co. Ltd., Beijing, China) that uses fused deposition modeling (FDM) technology and ABS thermoplastic material. The print settings were of fine quality, 99% internal fill, and 0.2 mm layer thickness.

The L7 vertebra and sacrum were joined by flexible filaments that aimed to promote mobility of the intervertebral joint, mimicking the flexibility of the spine that occurs when dogs are in different positions for insertion of the needle in the lumbosacral epidural space and injection of a local anesthetic.

### *Students and educational context*

The students and owners of the animals used in the clinical trial were duly informed about the study’s objectives, risks, benefits, and other implications and signed the respective informed consent form and authorization for the surgical and anesthetic procedures.

### *Study design*

Twenty students without distinction of gender, age or grades were randomly selected and assigned to control (n = 10) and 3DMEA (n = 10) groups. Veterinary medicine students who had already completed veterinary anesthesiology coursework but had never performed an epidural procedure were included in the study.

The 20 students in both groups one by one watched once a 3 minutes video showing the epidural technique step by step (Epidural continua en perro – Universidad Complutense de Madrid). After the video, the student could ask the veterinarian anesthesiologist any questions about the technique for 20 minutes, and in this same period, students in the 3DMEA group were allowed to train with the model.

With the model and the aid of the veterinarian anesthesiologist, the students trained the positioning of the animal, with the movement of the pelvis and the epidural technique, with the palpation of the anatomical structures and the correct positioning of the needle. The 3DMEA role simulated only the bones of the lumbosacral region to show students the palpation regions. This model did not have any type of material covering the bones. Thereafter, the students were allowed to perform the procedure in a live animal under supervision of the veterinarian anesthesiologist.

After a thorough surgical preparation, the landmarks for 22 gauge needle placement were identified in dogs by the students. The iliac prominences on either side of the spine were palpated by using the thumb and middle finger of one hand. The spinous process of the L7 vertebra was located with the index finger. The needle was placed correctly on the midline and caudal to the L7 spinous process, and is inserted until a distinct popping sensation is felt as the needle point penetrates the interarcuate ligament. The epidural space could be identified by the loss-of-resistance test, using a saline-filled syringe. The local anesthetic solution was injected through a spinal needle as a single dose.

### *Clinical trial*

The study included 20 male dogs, with mean ± standard deviation (range), aged 3.5 ± 2.3 (1.0–9.0) years, mean body mass of 12.1 ± 3.2 (8.0–18.0) kg, and body condition score 4-6 (scale 3-8)^21^. All animals used in the clinical trial were classified as healthy according to the American Society of Anesthesiologists as ASA I (normal clinical and laboratory examination findings). The pre-anesthetic laboratory tests performed included: Complete hemogram, Alkaline Phosphatase, Urea, Creatinine and Alanine Aminotransferase. The food and water were withheld for 12 and 2 hours, respectively, before anesthesia. Prior to the procedure, the animals were premedicated with acepromazine 0.05 mg kg^-1^ and tramadol 4 mg kg^-1^(50 mg ml^-1^,) intramuscularly, and then the hair from the lumbosacral region was clipped.

It was performed an anesthetic induction with intravenous propofol 5 mg kg^-1^in all animals. Posteriorly, the animals received tracheal intubation and were maintained by inhalation anesthesia with isoflurane in a rate of 1.2V%, in plane that allows the animal to stay anesthetized but responsive to nociceptive stimuli in pelvic limbs. When observed that the animal was in the ideal anesthetic plane, the dogs were positioned in sternal recumbency and had their pelvic limbs pulled cranially (sphinx down position) to enlarge the lumbosacral space.

Epidural anesthesia consisted of lidocaine hydrochloride 2% with epinephrine 0.002% 0.25 ml kg^-1^. The successful blockade was observed through one of these methods: identification of the epidural space by confirmation of ‘pop’ sensation, by the ‘hanging drop’ technique and lack of resistance to injection, besides the confirmation of the absence of nociceptive stimuli in pelvic limbs. The students were entitled to three attempts to perform the blockade; if the student failed all three, an error in the technique was identified. After the epidural injection was performed, the animals were prepared for orchiectomy.

The animals were monitored with the aid of a multiparameter device with the following parameters: heart rate (HR), respiratory rate (*f*_R_), non-invasive oscillometric arterial blood pressure (ABP), hemoglobin oxygen saturation (SpO_2_), and esophageal temperature (T). Monitoring began at the preanesthetic examination, was repeated at 5 minutes intervals, and continued until the end of the surgical procedure.

The success of epidural anesthesia was confirmed by the following parameters: 1. Tail relaxation; 2. Absence of nociceptive signal to phalangeal clamping in the pelvic limbs; and 3. Stability of HR, ABP, and *f*_R_ parameters (without changes > 20% of baseline values) during the incision.

If one or more of the above parameters were changed, the blocking was considered unsatisfactory and the epidural was considered “unsuccessful.” If the animals presented pain-related signs, analgesic rescue was performed with the intravenous administration of tramadol 5 mg kg^-1^. After the procedure, all the animals received meloxicam 0.1 mg kg^-1^, remained under observation for six hours and then discharged.

### *Assessment questionnaire*

**Figure 1:**
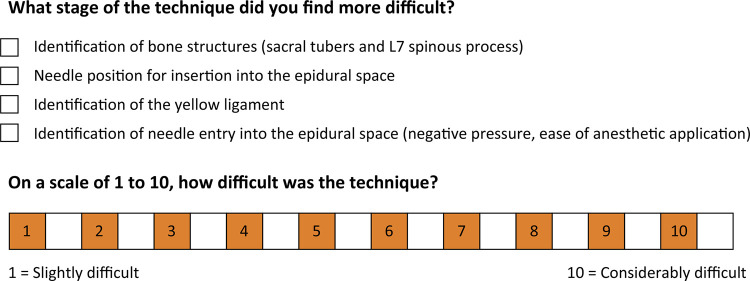
Assessment questionnaire. Through the questionnaire, the students assessed the main difficulty during the technique, giving it a degree of difficulty in the procedure, giving a grade ranging from 1 to 10.

At the end of each procedure, all students completed a questionnaire that assessed the main difficulty encountered during the technique and the degree of difficulty attributed to its accomplishment (Fig. 1).

### *Statistical analysis*

Fisher’s exact test was used with a 5% significance level to compare the number of successful epidurals between the control and 3DMEA groups. Student *t* test at 5% significance was first used to compare the means of scores attributed to epidural degree of difficulty between groups.

## Results

The image scanning and editing processes performed of the bone pieces allowed a consistent reproduction of the dog’s skeleton lumbosacral region. The 3DMEA ensured accurate visualization of the L7 spinous process and iliac prominences.

**Figure 2:**
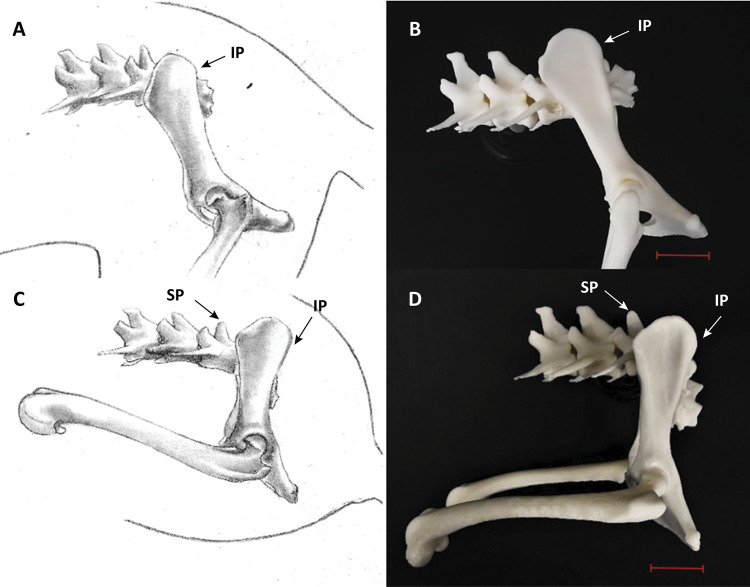
Illustration (A and C) and image of MAP3D (B and D) in the standing position and sternal recumbency, respectively – lateral view. Representation of the animal in standing position (A and B) and sternal decubitus (C and D). IP: iliac prominence; SP: spinous process of L7. Scale, 2 cm.

**Figure 3:**
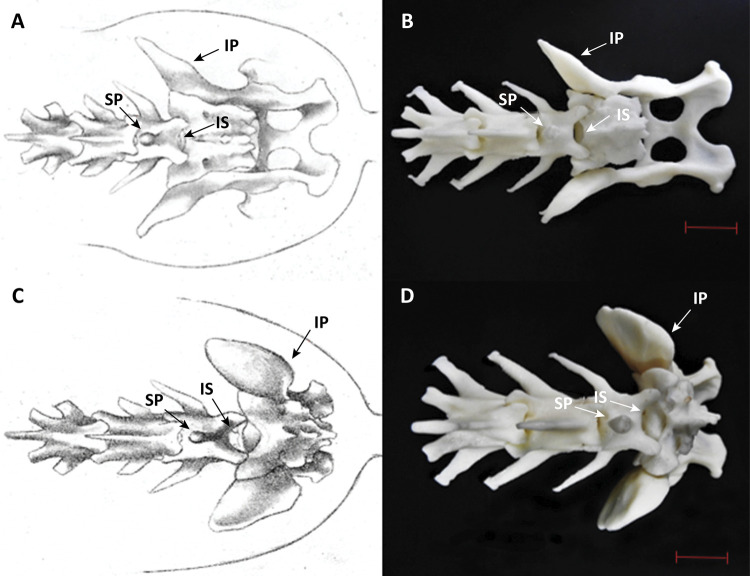
Illustration (A and C) and image of MAP3D (B and D) in the standing position and sternal recumbency, respectively – dorsal view. Representation of the animal in standing position (A and B) and sternal decubitus (C and D). IP: iliac prominence; IS: intervertebral lumbosacral space; SP: spinous process of L7. Scale, 2 cm.

The mechanism of attachment between the L7 and 3DMEA sacral bone allowed the increase and decrease of the intervertebral space during flexion and rest, respectively (Figs. 2 and 3). Information on creation and printing times as well as the quantity of material used to manufacture 3DMEA and the final costs of printing are described in Table 1.

**Table 1:** Creation time, production time, and total individual costs of MAP3D.

PIECE	CREATION TIME (h)	PRINT TIME (h)	MATERIAL USED (g)	COST (US$)
Vertebra L5	1.5	1.7	12.89	0.37
Vertebra L6	1.5	1.4	10.57	0.30
Vertebra L7	1.5	1.4	10.50	0.30
Sacrum	2.5	2	17.63	0.50
Pelvis	2.0	5.4	45.57	1.30
**Total**	**9.0**	**11.9**	**97.16**	**2.77**

(h) - Hours; (g) - Grams; (US$) – US Dollars

In determining 3DMEA costs, a cost of US $21.96 for each 1000 g of acrylonitrile butadiene styrene (ABS) filament was considered, and the cost of the raw material corresponded to 70% of the total cost. The raw material costs used for printing of supports, depreciation of equipment (scanner and 3D printer costs), and consumption of electric energy corresponded to the remaining 30%.

Among the 20 students participating in the study, 45% (9/20) successfully performed the epidural technique, six in the 3DMEA group and three in the control group. Despite the numerical difference, there was no significant intergroup difference (p = 0.3698) (Table 2).

**Table 2:** Frequency values of canine lumbosacral epidural anesthesia (CLEA) success by group and mean and standard deviation of epidural degree of difficulty execution of control and 3DMEA groups. The grades attributed to the degree of difficulty were arranged on a scale of 1 to 10.

Parameters	Control	3DMEA
CLEA f (n = 10/group)	3^a^	6^a^
Degree of difficulty (Mean ± SD)	6.4 ± 1.7^b^	3.4 ± 2.0 ^c^

When evaluating the means of scores attributed to the degree of difficulty between the two groups, the 3DMEA group had a mean of 3.4, while the control group had a mean of 6.4, which in practice represented a significant intergroup difference (p = 0.0037).

The students who achieved epidural success but who did not use 3DMEA identified palpation of the bone structures (iliac prominences and L7 spinous process) as the main difficulty (4/10). In the 3DMEA group, the main difficulty was identifying the ligamentum flavum (5/10). In addition, no students in the 3DMEA group indicated difficulty palpating the bone structures.

## Discussion

None of the 20 animals had any complication or needed analgesic rescue, because everyone received the epidural block before surgery. Although its use in the analgesic rescue was unnecessary, tramadol use in pre-anesthetic medication provided the necessary analgesic support for the procedures. Its use has shown to be successful in the acute pain control, with effective relief of post-surgical discomfort in cases of mild and moderate pain^22,23^.

Another cause that explains the absence of analgesic rescue is the time effect of epidural anesthesia (1-2h) that exceeded the surgery time (10-20min). The epidural anesthesia was also performed by the veterinary anesthesiologist in the animals that the students failed to anesthetize at the test. As a general rule, the addition of a vasoconstrictor to a local anesthetic agent, such as epinephrine, allows for decreased local perfusion, delayed rate of vascular absorption of local anesthetic, and therefore increased intensity and prolonged anesthetic activity^24^.

The total print time of the 3DMEA parts was 11.9 hours, with 97.16 g of ABS filament being spent. Our results were similar to other research that reported a printing time of 23.6 hours to produce a 140 g bovine femur^17^, and 4.5 hours and 5.5 hours for the production of 26 g and 28 g of human upper and lower jaw models, respectively^25^.

The total production cost of 3DMEA was US$2.77, which corroborates similar values among other studies^17,25^. The use of FDM printers allow 3D models to be produced for clinical cases at a cost that rarely exceeds US $5^19^.

3DMEA presented compatibility with the size of the animals used in the study and was able to accurately reproduce structures such as L7 spinous process and iliac prominences that were essential for the technique. Our study corroborates with a study that reported the ability to reproduce anatomical structures reliably when studying the reproduction of pelvic fractures in dogs using 3D printing^19^.

At the time that 3DMEA was flexed to simulate the animal’s articulation, the student had the perception of the alignment between the tubers and L7 spinous process and the increase of the intervertebral space as well as its direct correlation with the subject’s positioning during the epidural anesthesia. This increase facilitated the recognition of the exact point for needle insertion, guaranteeing greater safety and increasing the chances of successful blockade execution^26^.

The students who used 3DMEA in their previous training had a greater number of blockade successes, but this did not reflect significant results in the different groups. We can supposedly attribute this fact to the reduced number of students in each group^8^.

In addition, a significant intergroup difference was observed in the mean scores attributed to the degree of difficulty performing the epidural anesthesia. The lower mean of the 3DMEA group indicated that the students who used the model felt less difficulty performing the technique than those in the other group. These results corroborate with the study, which reported that the students who studied equine phalanges anatomy using 3D models presented less difficulty solving the proposed evaluation than students who studied through 3D virtual images or 2D images^18^.

Using a 3D model prior to the training of thoracic epidural anesthesia in humans, it was observed that using a model promoted a greater number of correct answers than the traditional method^8^. Additionally, the use of 3D models promoted a significant increase in the spatial comprehension of complex anatomical structures^18^. Thus, we verified that the previous training with 3D models increased the probability of the recognition of anatomical structures and consequently greater safety with the student in the blockade.

Not all students in the 3DMEA group displayed epidural success. The main obstacle cited by the unsuccessful students was related to identification of the ligamentum flavum, characterized by a popping sensation caused by the needle crossing it. Reported cases indicate that the identification of characteristic signals of successful blockade may not occur due to factors that depend on the anatomy and physiology of individual animals^4^.

The thickness of the ligamentum flavum in animals may vary by age, which may have hampered their identification during the technique^27^. Supposedly, the age variation of the animals used in our study added to the fact that 3DMEA did not intend to reproduce the ligamentum flavum, caused this difficulty for the students regardless of correctness or errors in the blockade execution.

An interesting fact to note was that few students in the control group indicated the identification of ligamentum flavum as the main difficulty since this was the main obstacle to be reported by the 3DMEA group. This fact may have occurred due to the great difficulty that the control group students had in relation to identifying anatomical structures, the main obstacle observed by the group.

Regarding the execution of the technique, the spinal cord in dogs ends at the L7 vertebra forming the medullary cone, at the end of the cone is a cord formed by the pia mater and the terminal filament called “cauda equina” before the lumbosacral intervertebral space where the epidural anesthesia is performed^28^. This information was given to the students during the video exhibition, which in practice made them calmer because they were confident that introducing the needle to the blockade would not cause lesions in the spinal cord.

However, as much as the video helps in the relation of teaching and learning of students, its isolated presentation in the present study was insufficient to improve the indexes of success and to decrease the degree of difficulty performing the epidural anesthesia. Our findings support the statement that visual and auditory stimuli do not replace practical experience in the acquisition of clinical skills^29^.

The great challenge in creating epidural anesthesia models is accurately simulating all of their anatomical structures, such as skin, subcutaneous fat, ligaments, and bones. This difficulty creates obstacles in the training of epidural anesthesia and compromises its success^30^.

Despite the promising results to date, 3DMEA presented limitations. The absence of structures that mimic the ligamentum flavum and the negative pressure were obstacles to a greater number of successes in the technique. The lack of perception of characteristic signals confirming needle entry into the epidural space as well as the absence of negative pressure and lack of perception of plunger resistance are limitations to epidural success^4,26^.

Although the small sample size may have made statistical analysis difficult, this was enough to provide a more accurate learning analysis of students’ performance during the test, which allowed observing a better performance in the 3DMEA group, where the students showed more confidence in palpation of anatomical structures than the other group.

## Conclusions

The use of 3DMEA before epidural anesthesia by inexperienced students resulted in greater efficiency in the execution of the technique in live dogs subjected to orchiectomy since it facilitated the anatomical identification of the L7 spinous process and iliac prominences, being effective in the teaching and learning process prior to epidural in live animals. Thus, its potential use in teaching was demonstrated. However, further studies with a greater number of animals and students are necessary to validate our findings.
